# The Management of a Giant Convexity en Plaque Anaplastic Meningioma with Gerstmann Syndrome: A Case Report of Surgical Outcomes in a 76-Year-Old Male

**DOI:** 10.3390/diagnostics14222566

**Published:** 2024-11-15

**Authors:** Corneliu Toader, Felix Mircea Brehar, Mugurel Petrinel Radoi, Matei Serban, Razvan-Adrian Covache-Busuioc, Ghaith S. Aljboor, Radu M. Gorgan

**Affiliations:** 1 Department of Neurosurgery “Carol Davila”, University of Medicine and Pharmacy, 050474 Bucharest, Romania; matei.serban2021@stud.umfcd.ro (M.S.); corneliu.toader@umfcd.ro (C.T.); razvan-adrian.covache-busuioc0720@stud.umfcd.ro (R.-A.C.-B.); ghaith-saleh-radi.aljboor@drd.umfcd.ro (G.S.A.); radugorgan@umfcd.ro (R.M.G.); 2 Department of Vascular Neurosurgery, National Institute of Neurology and Neurovascular Diseases, 077160 Bucharest, Romania; 3 Department of Neurosurgery, Clinical Emergency Hospital “Bagdasar-Arseni”, 041915 Bucharest, Romania

**Keywords:** giant en plaque meningioma, Gerstmann syndrome, anaplastic meningioma, convexity meningioma, superior sagittal sinus infiltration, peritumoral brain edema, Simpson grade resection, hypervascular brain tumor

## Abstract

Background: This case report highlights a rare presentation of a giant convexity en plaque anaplastic meningioma, located in the left frontoparietal parasagittal region, infiltrating the superior sagittal sinus, and associated with Gerstmann syndrome. This study aims to explore the clinical challenges, surgical management, and potential reversibility of neurological deficits induced by the tumor, including those characteristic of Gerstmann syndrome. Methods: A 76-year-old male patient presented with a history of worsening expressive aphasia and cognitive impairments, culminating in a generalized seizure. Preoperative imaging confirmed a 4 × 6 cm highly vascularized tumor with significant peritumoral edema. The patient underwent near-total resection of the tumor, aiming for a Simpson grade 2 resection, while managing hypervascularity and brain edema. Histological analysis confirmed the diagnosis of anaplastic meningioma (WHO Grade III), showing features such as necrosis, brain invasion, and high mitotic activity. Results: Post-surgical follow-up demonstrated significant improvement in the patient’s neurological deficits, particularly in expressive language and cognitive function, suggesting a potential reversal of Gerstmann syndrome. Postoperative imaging revealed a moderate degree of cerebral collapse and absence of contrast leakage. Two-month follow-up confirmed no recurrence of neurological deficits. Conclusions: This case emphasizes the complexity of managing giant convexity en plaque anaplastic meningiomas, particularly when associated with Gerstmann syndrome. Surgical resection, despite the challenges posed by tumor size, hypervascularity, and peritumoral edema, can lead to significant neurological recovery, highlighting the potential reversibility of tumor-induced Gerstmann syndrome.

## 1. Introduction

The 2021 World Health Organization (WHO) classification of meningiomas provides a taxonomy with 15 histopathological subtypes that fall under one type [1]. Each subtype is further subdivided into three grades of malignancy depending on specific features: grade 1 comprises the most common subtypes like meningothelial, fibrous, and transitional meningiomas that tend to be benign; grade 2 encompasses chordoid and clear cell meningiomas, which have a higher risk of recurrence; while grade 3 anaplastic (malignant) meningiomas, which tend to recur often, have greater risks and are associated with a worse prognosis than their counterparts.

Meningiomas constitute the most common non-malignant brain tumor, comprising 37.6% of all tumors according to Ostrom et al. [2].

Meningiomas become more prevalent as people become older, with an average diagnosis age of 66 years. Their incidence rate among these patients stands at 18.69 per 100,000 individuals while among 0–19-year-old individuals it drops significantly to 0.16 per 100,000 individuals. Meningiomas account for 43.6% of all central nervous system (CNS) tumors among patients aged 40 or above, while 15.6% appear between 15 and 39 years old, and 1.7% among individuals of 0–14 years; females have a higher prevalence rate, with both benign and malign types’ ratios being 2.33 and 1.12, respectively [3]. Meningiomas typically grow with an en masse growth pattern, characterized by an infiltrative, globular mass. En plaque meningiomas are an unusual subtype distinguished by an extensive carpet-like growth pattern with dural and bone invasion; these tumors tend to occur in the spheno-orbital region and frequently exhibit signs of hyperostosis—an abnormal thickening of bone tissue [4].

Meningioma size classification follows the guidelines established by Russell et al., in which small meningiomas are defined as those measuring less than 2 cm in diameter, medium-sized meningiomas range between 2 to 4 cm in size, and large meningiomas can reach up to 4.9 cm diameter [5].

Meningiomas present with different clinical signs depending on their size and anatomical location, with most patients experiencing headaches (48.2%). Individuals with convexity meningiomas typically exhibited cerebral dysfunction while 38.9% of individuals with skull base meningiomas demonstrated cranial nerve deficits [6]. On the other hand, Gerstmann syndrome (GS) is a cognitive disorder characterized by neurological deficits in the left hemisphere of the brain [7]. These deficits typically impact the left angular gyrus as it transitions into the second occipital convolution and could include damage from stroke, brain tumors exerting pressure, or paroxysmal Gerstmann syndrome arising as part of epilepsy [8].

Although GS is frequently associated with cerebrovascular events or degenerative neurological conditions, it has been documented in rare cases involving neoplastic lesions, particularly those affecting the dominant parietal cortex. Tumor-induced GS is exceedingly rare and represents a diagnostic challenge, as its manifestations can overlap with other cognitive impairments associated with intracranial tumors. The recent literature has documented only a limited number of cases in which intracranial tumors, such as glioblastomas and atypical meningiomas, have produced GS, and fewer still in which anaplastic meningiomas were implicated [9,10]. The case presented here highlights this unusual association, as it describes a 76-year-old male with a giant convexity en plaque anaplastic meningioma, infiltrating the superior sagittal sinus and resulting in GS. This report aims to expand the existing literature on tumor-induced GS by detailing the surgical complexities and patient outcomes observed in managing aggressive, highly vascularized meningiomas in critical neuroanatomical regions. By exploring these aspects, this study underscores the relevance of GS as a potential paraneoplastic syndrome in certain intracranial tumor profiles, and it provides insights into the reversibility of tumor-induced cognitive impairments post-surgical intervention.

## 2. Case Presentation

A 76-year-old male patient was admitted to our clinic following a generalized grand mal seizure that occurred three days prior, in what was previously a state of apparent good health. The patient has a history of progressively worsening expressive language disturbances and moderate cognitive impairments over the past six months.

Upon neurological examination, the patient presented with a right-sided pyramidal syndrome, expressive aphasia, and features consistent with GS in the dominant hemisphere. Given these findings, a series of neuropsychological assessments were conducted to confirm the diagnosis of GS, targeting its four primary features: left–right disorientation, finger agnosia, agraphia, and acalculia.

The patient exhibited significant left–right disorientation, struggling to correctly identify left and right on both his body and external objects. This disorientation, frequently associated with left parietal lobe dysfunction, is a characteristic indicator of GS.

Finger agnosia was evident during tasks in which the patient was asked to identify specific fingers on command and to recognize fingers when touched by the examiner. His responses were inconsistent and often incorrect, confirming a classic symptom of GS: impaired finger recognition.

Writing ability was assessed through dictated words and sentences, revealing fragmented and incoherent writing with frequent spelling and syntax errors. This disruption in written language processing, characteristic of agraphia, further supported the diagnosis.

Finally, arithmetic ability was evaluated through basic addition and subtraction problems, in which the patient struggled with even simple calculations, showing deficits in numerical cognition indicative of acalculia.

**Figure 1:**
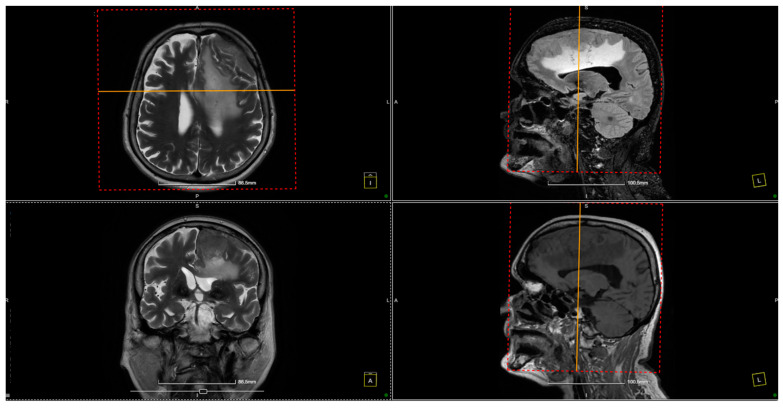
Preoperative MRI. The preoperative MRI scans display a large, convexity en plaque meningioma in the left frontoparietal region, extending toward the parasagittal area. The tumor exhibits homogenous enhancement on T2-weighted images, indicating a highly vascular lesion consistent with meningiomas. The tumor’s dimensions are approximately 4 × 6 cm, and it infiltrates the dura mater, extending deeply toward the paraventricular area. This infiltration reaches the superior sagittal sinus, posing challenges for surgical resection due to the involvement of this critical venous structure. The dashed frames indicate the area of interest, while the yellow lines mark intersecting planes across axial, sagittal, and coronal views, facilitating anatomical correlation and spatial orientation for surgical planning.

**Figure 2:**
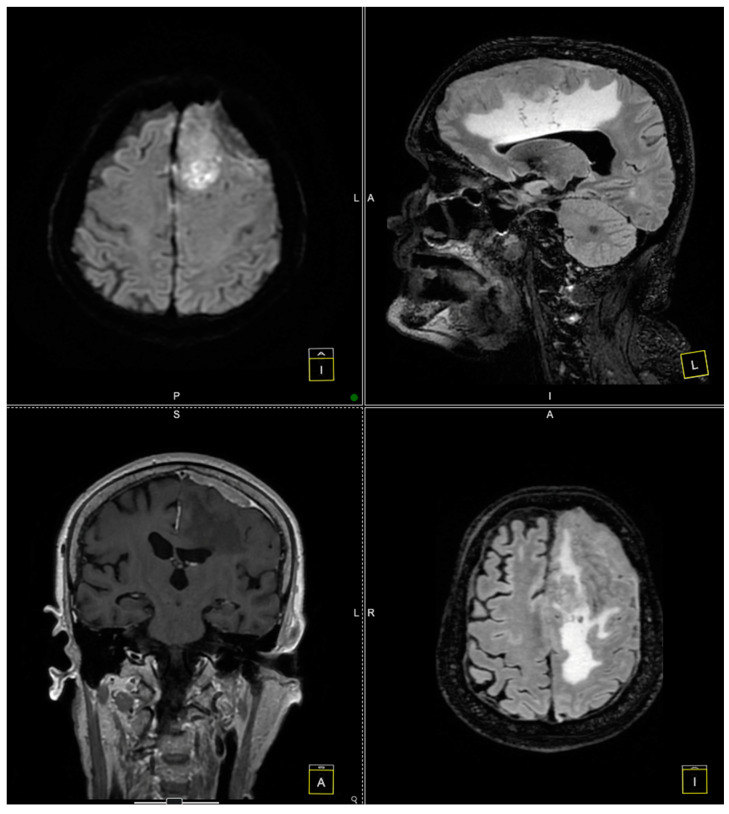
Preoperative MRI. The preoperative MRI scans illustrate a large, homogeneously enhancing convexity en plaque meningioma located in the left frontoparietal region. In these images, the tumor’s infiltration of the dura mater and partial involvement of the superior sagittal sinus are evident, posing additional complexity for surgical resection.

Further evaluation with contrast-enhanced MRI confirmed the presence of a tumoral mass, which was isointense with brain parenchyma on T1-weighted images and moderately hyperintense on T2-weighted images, with intense and homogeneous enhancement following contrast administration. Notable peritumoral cerebral edema was observed, extending to the interhemispheric region and the corpus callosum. The lesion appeared suggestive of a meningioma measuring 4 × 6 cm. The remainder of the brain appeared normal on T1- and T2-weighted sequences, as well as after contrast administration (Figure 1 and Figure 2).

**Figure 3:**
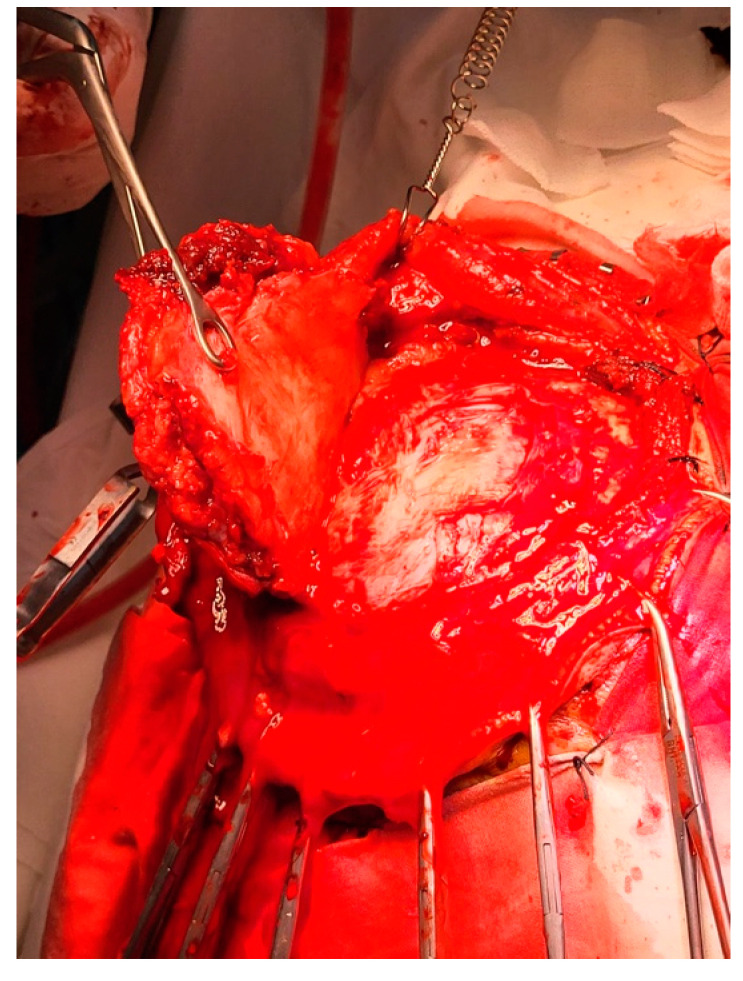
Left frontoparietal parasagittal region.

**Figure 4:**
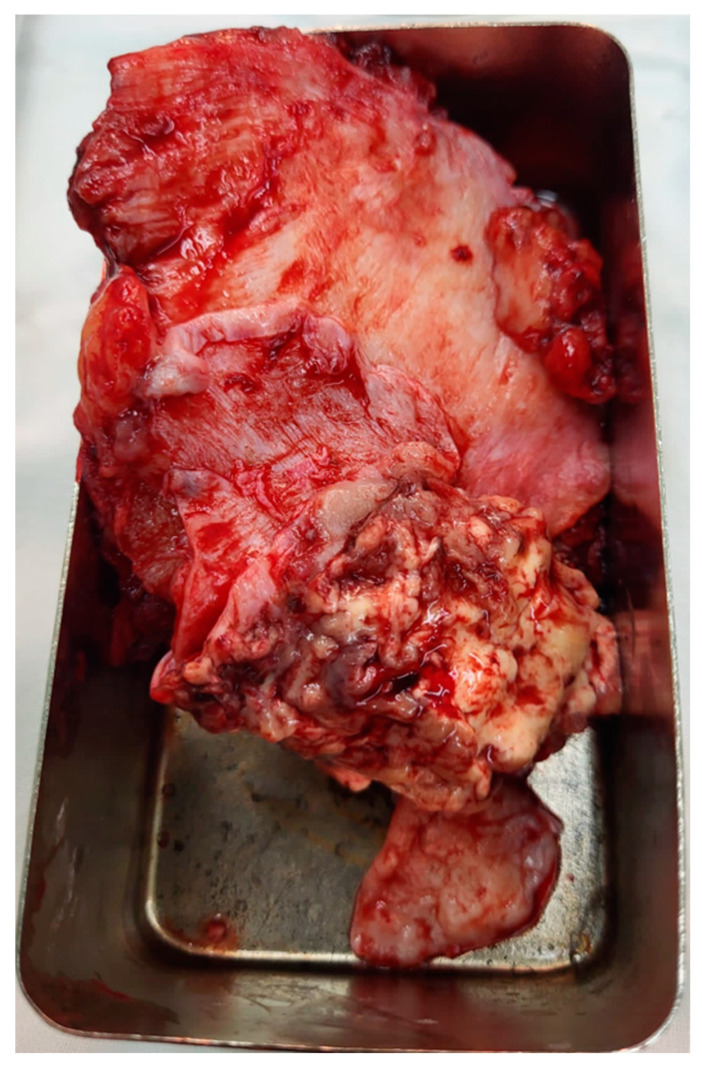
Macroscopic view of the tumor.

The infiltrated dura mater was completely resected along with a large, firm, grayish meningioma located in the left frontoparietal parasagittal region (Figure 3). The tumor, measuring 4 × 6 cm at its maximum diameter, was highly vascularized and extended deeply toward the paraventricular area, infiltrating the wall of the superior sagittal sinus (SSS) while sparing the adjacent brain parenchyma and maintaining normal vascular structures (Figure 4). The tumor was resected to a near-total extent macroscopically. A moderate degree of cerebral collapse was noted. Hemostasis was achieved using electrocoagulation and Surgicel. A dural defect resulted from the removal of the infiltrated dura, and the tumor-infiltrated bone flap was also removed. The scalp was then sutured in anatomical layers, and the wound was dressed. We estimate that Simpson grade 2 was achieved after surgery.

In this case, cerebral collapse was managed through careful monitoring of the intracranial pressure (ICP) and implementing strategies to maintain adequate cerebral perfusion. Postoperative care included positioning adjustments, fluid management, and the administration of osmotic agents to reduce intracranial pressure when needed. Additionally, follow-up imaging was conducted to monitor cerebral collapse progression and ensure no new areas of infarction or ischemia. We also emphasize the role of physical and cognitive rehabilitation in supporting functional recovery, as early mobilization and therapy can mitigate the effects of cerebral collapse on neurological function.

**Figure 5:**
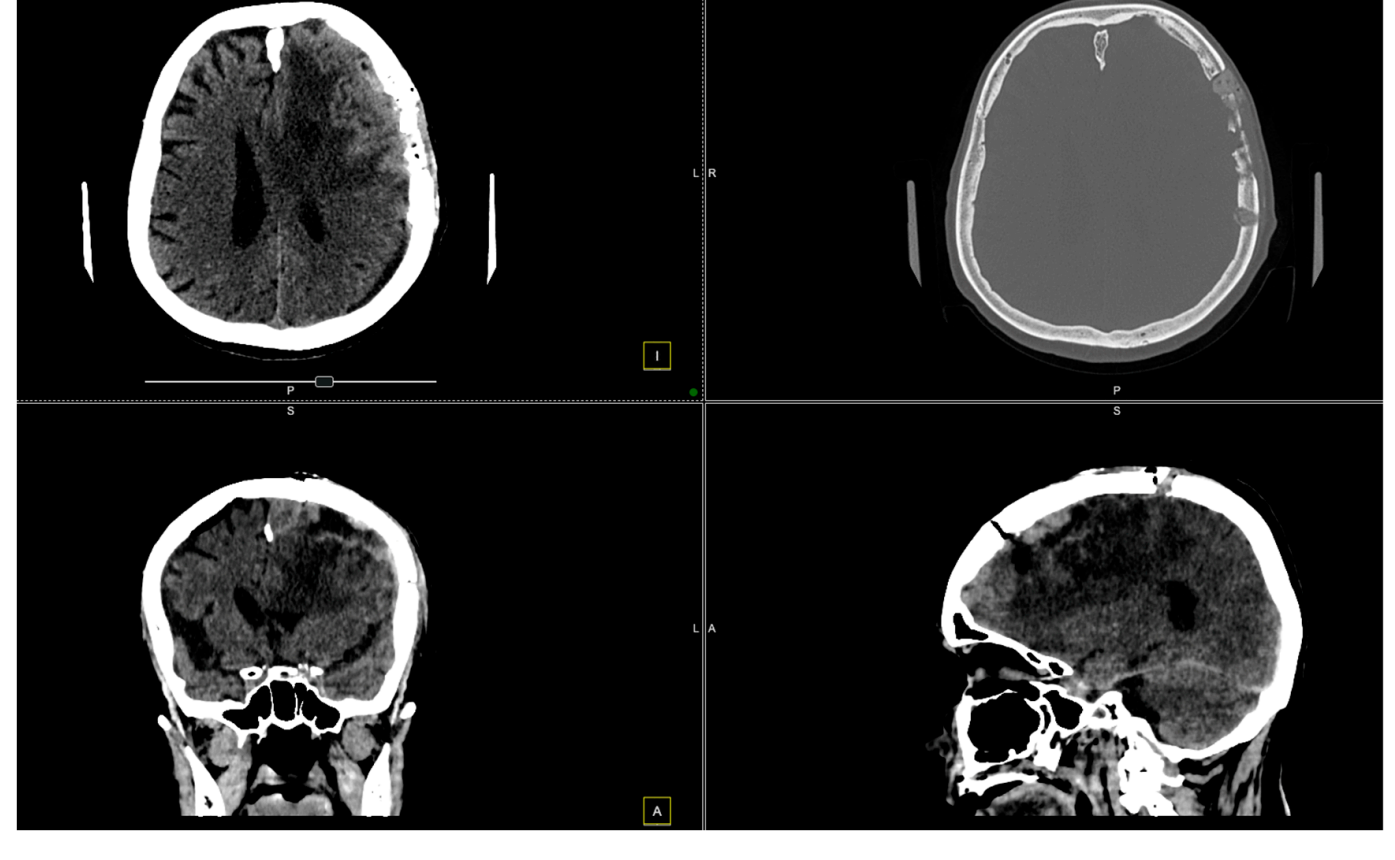
CT scan conducted 7 days post-surgery.

**Figure 6:**
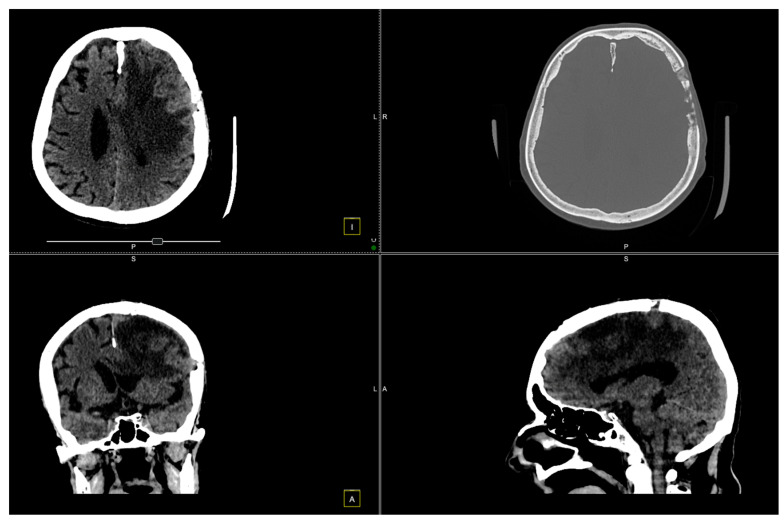
CT scan conducted 14 days after the surgery.

The postoperative course was favorable, with significant neurological improvement observed (Figure 5 and Figure 6).

**Figure 7:**
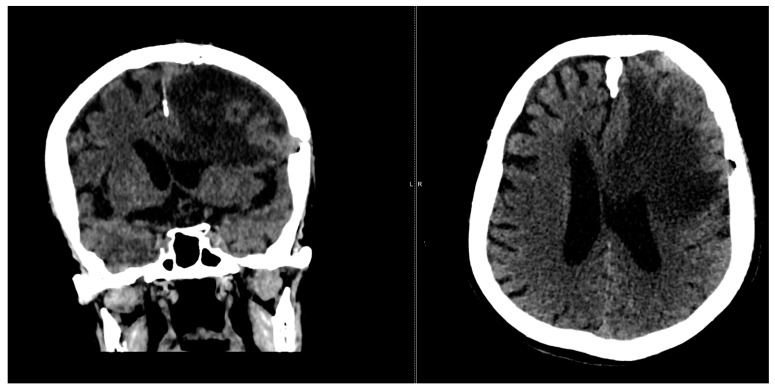
CT scan shows left paramedian frontal hypodensity, without contrast outlet.

At the 2-month follow-up check, we conducted another CT scan (Figure 7).

**Figure 8:**
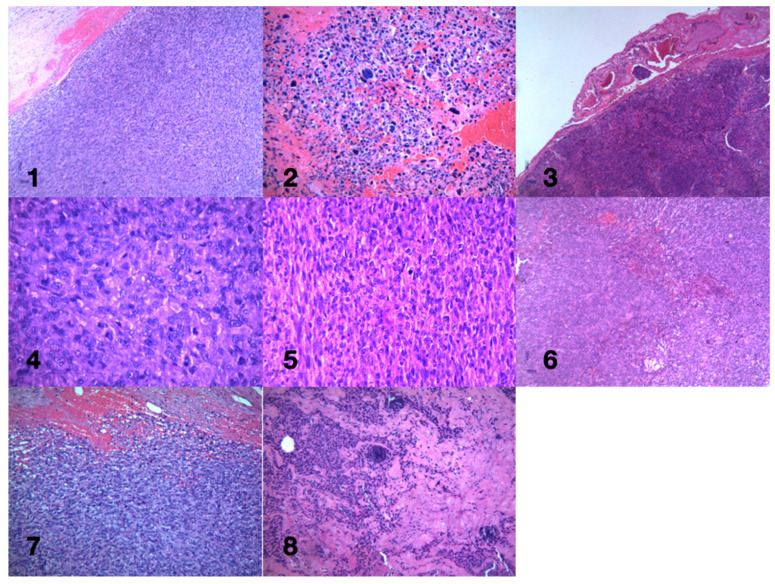
The histological images illustrate key features of anaplastic meningioma (WHO Grade III), including extensive necrosis, brain and vascular invasion, and high cellular atypia. Tumor cells show pleomorphism, hyperchromatic nuclei, and high mitotic activity, indicative of aggressive malignancy. Hemorrhagic areas highlight rapid tumor growth, while classic meningioma structures like meningothelial whorls and psammoma bodies are also present. These findings emphasize the highly invasive and malignant nature of anaplastic meningiomas. Anaplastic meningiomas, classified as WHO Grade III, exhibit aggressive features such as necrosis, brain invasion, and overtly malignant cytomorphology. The histological sections (Images **1**–**3**,**6**,**7**) highlight key pathological characteristics, including extensive necrosis, brain parenchyma invasion, and cellular pleomorphism. In particular, Image **1** shows marked necrosis and brain invasion, a hallmark of aggressive meningioma behavior, while Image **2** reveals cytomorphology resembling high-grade sarcoma, with pleomorphic, hyperchromatic nuclei and abundant mitotic figures. Vascular invasion, visible in Image **3**, is another critical feature of high-grade meningiomas, increasing metastatic potential. Poor differentiation and hemorrhagic areas (Images **4**,**8**) underscore the malignancy, with irregular tumor cells and compromised vascular support leading to hemorrhage. Classic meningioma features, such as whorls of meningothelial cells and psammoma bodies, are also observed (Image **5**), which, while typical of benign forms, can still be present in more aggressive variants. These images, stained with HE, provide a comprehensive view of the aggressive, infiltrative, and poorly differentiated nature of anaplastic meningiomas.

Due to the high recurrence risk associated with WHO Grade III tumors (Figure 8), routine follow-up with imaging is essential to promptly detect any signs of regrowth.

Our follow-up approach includes periodic MRI scans and comprehensive neurological assessments to track any potential recurrence and monitor the patient’s cognitive and neurological status over time. This strategy not only aids in early intervention should the tumor recur but also allows us to assess changes related to Gerstmann syndrome symptoms or other neurological functions.

By incorporating regular follow-up as a key component of post-treatment care, we aim to support better long-term outcomes for patients with aggressive meningiomas.

## 3. Discussion

Giant intracranial meningiomas frequently present with peritumoral brain edema (PBE), a factor that worsens prognosis and complicates surgical outcomes. Despite extensive research, the exact causes of PBE remain unclear [11]. Our patient experienced substantial brain edema, which improved post-surgery, as demonstrated in follow-up CT images. Preoperative imaging of skull base meningiomas often reveals distinct fingerlike projections of edema in the bifrontal white matter, a potential indicator of poorer postoperative outcomes [12].

Meningiomas tend to be slow-growing lesions, with an average annual growth rate of approximately 2.4 mm [13]. This gradual progression allows for substantial expansion before symptoms appear. Hyperostosis, commonly observed in en plaque meningiomas (MEPs), can compress nearby structures, leading to clinical symptoms [14].

MEP, commonly found on convexity sites, recurs frequently at approximately 25–50% (for sphenoid wing meningiomas) rates and remains under-documented; partial or subtotal resection has often been employed to manage and relieve symptoms; yet complete removal remains less commonly documented [15,16]. The resection of a giant intracranial meningioma (GIM), regardless of its location, presents numerous challenges. These include its considerable size, hypervascularity, PBE, brain tension, and complexity of surrounding neurovascular structures—not to mention difficulty of adequate visualization [17]. Brain edema often becomes an impediment during GIM surgery, with its presence limiting the surgical corridor and further complicating procedures considerably, due to tension in the brain tissue or secondary edema that restricts the surgical corridor, combined with mass effect or midline shift further complicating procedures significantly [8]. Managing blood loss is a critical concern when operating on a GIM; therefore, Bendszus et al. demonstrated how complete embolization effectively helps manage intraoperative blood loss during GIM surgery [12]. Considering the age of our patient, and in order to ensure good quality of life and outcome, Simpson grade 2 was achieved, which translates to an approximate 19% recurrence rate [7,18].

Although contralateral hemisensory loss due to lesions of the postcentral somatosensory cortex is expected, lesions in the dominant parietal lobe can also produce Gerstmann syndrome. This syndrome is classically defined by four clinical signs: left–right confusion, finger agnosia, agraphia, and acalculia; however, these rarely appear alone and often co-occur with other, less commonly observed findings such as neglect, inattention, aphasia apraxia disorders spatial orientation issues or difficulties, and word-finding difficulties [18]. In our case, resection of the meningioma provided symptom resolution. Recent studies on giant convexity en plaque anaplastic meningiomas, particularly those associated with Gerstmann syndrome, highlight the complexity and variability in presentation, diagnosis, and management (Table 1). Tumor sizes in these cases range significantly, with some reaching up to 9 cm in diameter, often located in critical regions such as the convexity and superior sagittal sinus. The presence of peritumoral edema is a common finding, contributing to the surgical challenges encountered in these cases. Tumor shapes vary from irregular to en plaque carpet-like growth patterns, frequently leading to significant brain compression and functional impairment. Histopathologically, most of these tumors are classified as anaplastic meningiomas (WHO Grade III), which are known for their aggressive behavior and high recurrence rates. The Ki-67 index, a marker for tumor proliferation, is often elevated (>10%) in these cases, further indicating the aggressive nature of the pathology. Surgical management often involves gross total resection, when possible, as reflected in Simpson grades I to IV across different studies. The postoperative outcomes are closely tied to the extent of resection, with higher Simpson grades correlating with increased recurrence risks. These findings underscore the importance of a multidisciplinary approach, combining advanced imaging, surgical techniques, and, in some cases, adjuvant therapies, to optimize patient outcomes in such rare and challenging cases.

The literature that discusses Gerstmann syndrome caused by tumoral compression is sparse, yet it represents proof that neurosurgery, in this case, can have results from symptom amelioration to complete symptom resolution [9,20,21]. Yang et al. present two patients who were diagnosed with left temporo-occipital tumors—specifically glioblastoma and meningioma, which resulted in the displacement of their left temporo-parieto-occipital cortex. Remarkably, both individuals displayed distinct forms of associative visual agnosia characterized by an inability to recognize smartphone icons; one in particular relied heavily on intact visuospatial awareness for smartphone use. One patient underwent gross total resection of meningioma for decompression of their temporo-parieto-occipital region, which led to complete resolution of their symptoms [5].

Recent reports underscore the challenges in diagnosing GS when caused by neoplastic lesions, as it more commonly arises from cerebrovascular accidents or neurodegenerative conditions [22]. While a limited number of studies have documented GS associated with brain tumors, these cases have primarily involved glioblastomas or atypical meningiomas situated in the dominant parietal cortex [23]. Unlike these more frequently reported cases, our patient’s GS symptoms were induced by a giant convexity en plaque anaplastic meningioma infiltrating the left frontoparietal region—a rarity both in location and in the pathological aggressiveness of the tumor.

Our case contributes to a nuanced understanding of tumor-induced GS by illustrating the direct link between focal compression by the tumor and the manifestation of GS’s characteristic symptoms: left–right disorientation, finger agnosia, agraphia, and acalculia. A recent study suggested that, when tumor-induced GS occurs, symptoms are typically less severe and may not fully match the classic tetrad of GS. In our case, however, the patient presented with a complete set of GS symptoms, underscoring the unique impact of the tumor’s size and infiltrative growth pattern on the dominant hemisphere’s parietal cortex.

Further distinguishing this case, recent findings have shown that the outcome of GS symptoms largely depends on the feasibility of complete resection and postoperative tumor control. Reports on similar cases document variable outcomes; for example, patients with subtotal resections often experience persistent or recurrent GS symptoms, whereas those with near-total or gross total resections, as achieved here, have demonstrated marked symptomatic improvement [24]. This aligns with our patient’s post-surgical recovery, where comprehensive tumor removal led to a near-complete reversal of GS symptoms, suggesting that aggressive surgical intervention may mitigate the cognitive impairments characteristic of GS, even in cases involving highly malignant meningiomas.

Our case also highlights the significance of ongoing monitoring given the aggressive behavior of anaplastic meningiomas. The literature indicates that patients with high Ki-67 indices, as seen in this case, are predisposed to recurrence, necessitating vigilant follow-up with imaging to promptly identify and manage any potential tumor regrowth [25,26]. In addition, emerging evidence suggests that adjuvant therapies, such as radiotherapy, may further reduce recurrence rates, particularly in high-grade meningiomas, though data on long-term GS symptom recurrence remain limited [27].

Ultimately, this case broadens the understanding of GS as a paraneoplastic syndrome that can present alongside high-grade, atypical meningiomas. By illustrating a rare, reversible presentation of tumor-induced GS, this case reinforces the importance of early intervention and targeted resection in optimizing cognitive and neurological outcomes. It also underscores the value of including GS in the differential diagnosis for patients presenting with isolated cognitive deficits, as timely surgical intervention may significantly improve their prognosis.

## 4. Conclusions

Our case report brings together two extreme rarities. A giant en plaque meningioma located in the left frontoparietal parasagittal region infiltrating the wall of the SSS, associated with Gerstmann syndrome. The surgical treatment of this patient was challenging in regard to the size of the meningioma, the age of our patient, PBE, and hypervascularity of the meningioma. The surgical intervention led to symptom amelioration regarding his neurological deficits, which stands as proof regarding the reversibility of tumor-induced Gerstmann syndrome.

## Figures and Tables

**Table 1:** Presents a comprehensive literature review on studies related to giant convexity en plaque anaplastic meningiomas, with a particular focus on those associated with Gerstmann syndrome. The table summarizes key patient characteristics, tumor features, and clinical outcomes from recent studies. Each row contains information on the number of patients involved, their age and sex, tumor size and location, presence of peritumoral edema, tumor shape, Simpson grade (extent of resection), subtype of pathology, and Ki-67 index (a marker for tumor proliferation).

Author(s) (Year)	Number of Patients	Age	Sex	Tumor Size	Location	Peritumoral Edema	Tumor Shape	Simpson Grade	Subtype of Pathology	Dichotomized Ki-67 Index
Ostrom QT et al. (2020) [2]	>100,000 (Statistical report)	N/A	Both	Various	CNS	N/A	N/A	N/A	Mixed tumor types	N/A
Louis DN et al. (2021) [1]	N/A	N/A	N/A	N/A	CNS	N/A	N/A	N/A	Anaplastic meningiomas (Grade III)	N/A
Ardila A (2020) [19]	2	65, 72	Male	Not measured	Left parietal	No	N/A	N/A	N/A	N/A
Elder TA et al. (2021) [4]	20	55–75	Both	2–6 cm	Skull base	Present	Carpet-like	I–III	Meningioma, en plaque	High (>10%)
Yang MJ et al. (2020) [5]	2	58, 62	Male	4–5 cm	Left parietal	Present	Irregular	II	Meningioma, glioblastoma	High (>10%)
Wu A et al. (2018) [6]	154	45–85	Both	2–8 cm	Convexity, skull base	Present	Lobulated	I–III	Meningioma	Mixed
Chotai S et al. (2022) [7]	N/A	N/A	N/A	N/A	CNS	N/A	N/A	I–IV	Mixed tumor types	N/A
Narayan V et al. (2018) [8]	36	50–75	Both	4–9 cm	Convexity	Present	Globular	II–IV	Anaplastic meningioma	High (>10%)
Rusconi E et al. (2010) [11]	3	60–70	Both	Not measured	Left parietal	No	N/A	N/A	N/A	N/A
Bendszus M et al. (2000) [12]	44	50–78	Both	3–9 cm	Convexity	Present	Lobulated	I–III	Anaplastic meningioma	High (>10%)

## Data Availability

The data presented in this study are available on request from the corresponding author.

## References

[1] Louis D.N., Perry A., Wesseling P., Brat D.J., Cree I.A., Figarella-Branger D., Hawkins C., Ng H.K., Pfister S.M., Reifenberger G. (2021). The 2021 WHO Classification of Tumors of the Central Nervous System: A summary. Neuro-Oncology.

[2] Ostrom Q.T., Patil N., Cioffi G., Waite K., Kruchko C., Barnholtz-Sloan J.S. (2020). CBTRUS Statistical Report: Primary Brain and Other Central Nervous System Tumors Diagnosed in the United States in 2013–2017. Neuro-Oncology.

[3] Ogasawara C., Philbrick B.D., Adamson D.C. (2021). Meningioma: A Review of Epidemiology, Pathology, Diagnosis, Treatment, and Future Directions. Biomedicines.

[4] Elder T.A., Yokoi H., Chugh A.J., Lagman C., Wu O., Wright C.H., Ray A., Bambakidis N. (2019). En Plaque Meningiomas: A Narrative Review. J. Neurol. Surg. Part B Skull Base.

[5] Yang M.J., Nail T.J., Winer J. (2020). Left Parietal Tumors Presenting with Smartphone Icon Visual Agnosia: Two Cases of a Modern Presentation of Gerstmann Syndrome. World Neurosurg..

[6] Wu A., Garcia M.A., Magill S.T., Chen W., Vasudevan H.N., Perry A., Theodosopoulos P.V., McDermott M.W., Braunstein S.E., Raleigh D.R. (2018). Presenting Symptoms and Prognostic Factors for Symptomatic Outcomes Following Resection of Meningioma. World Neurosurg..

[7] Chotai S., Schwartz T.H. (2022). The Simpson Grading: Is It Still Valid?. Cancers.

[8] Narayan V., Bir S.C., Mohammed N., Savardekar A.R., Patra D.P., Nanda A. (2018). Surgical Management of Giant Intracranial Meningioma: Operative Nuances, Challenges, and Outcome. World Neurosurg..

[9] Feller C., Kelly N. (2021). A-62 Case of Gerstmann’s Syndrome Due to Brain Tumor of Unknown Etiology. Arch. Clin. Neuropsychol..

[10] Gnanapavan S., Jaunmuktane Z., Baruteau K.P., Gnanasambandam S., Schmierer K. (2014). A rare presentation of atypical demyelination: Tumefactive multiple sclerosis causing Gerstmann’s syndrome. BMC Neurol..

[11] Rusconi E., Kleinschmidt A. (2011). Gerstmann‘s syndrome: Where does it come from and what does that tell us?. Futur. Neurol..

[12] Bendszus M., Rao G., Burger R., Schaller C., Scheinemann K., Warmuth-Metz M., Hofmann E., Schramm J., Roosen K., Solymosi L. (2000). Is There a Benefit of Preoperative Meningioma Embolization?. Neurosurgery.

[13] Kuratsu J.-I., Kochi M., Ushio Y. (2000). Incidence and clinical features of asymptomatic meningiomas. J. Neurosurg..

[14] Park H.K., Koh Y.C., Kang H.S., Lim S.D. (2006). Meningioma en plaque of parasagittal region presented with recurrent venous infarction. J Korean Neurosurg..

[15] Bouguila J., Khonsari R., Ayashi K., Neji N., Yacoub K., Besbes G. (2009). Méningiome en plaque frontal: Forme rare d’une tumeur commune!. Rev. Stomatol. Chir. Maxillo-Faciale.

[16] Simas N., Farias J. (2013). Sphenoid wing en plaque meningiomas: Surgical results and recurrence rates. Surg. Neurol. Int..

[17] Attia M., Umansky F., Paldor I., Dotan S., Shoshan Y., Spektor S. (2012). Giant anterior clinoidal meningiomas: Surgical technique and outcomes. J. Neurosurg..

[18] Simon M., Gousias K. (2024). Grading meningioma resections: The Simpson classification and beyond. Acta Neurochir..

[19] Ardila A. (2020). Gerstmann Syndrome. Curr. Neurol. Neurosci. Rep..

[20] Natteru P., Nair L.R., Luzardo G., Shaikh N. (2021). Meningeal Hemangiopericytoma Presenting as Pure Gerstmann Syndrome: A Double Rarity. Cureus.

[21] Kleinschmidt A., Rusconi E. (2011). Gerstmann Meets Geschwind: A crossing (or kissing) variant of a subcortical disconnection syndrome?. Neurosci..

[22] Aleo D., Elshaer Z., Pfnür A., Schuler P.J., Fontanella M.M., Wirtz C.R., Pala A., Coburger J. (2022). Evaluation of a Navigated 3D Ultrasound Integration for Brain Tumor Surgery: First Results of an Ongoing Prospective Study. Curr. Oncol..

[23] Ebel F., Greuter L., Licci M., Guzman R., Soleman J. (2021). Endoscopic and Endoscopically-Assisted Resection of Intraventricular Lesions Using a Neuroendoscopic Ultrasonic Aspirator. J. Clin. Med..

[24] Thellung S., Corsaro A., Bosio A.G., Zambito M., Barbieri F., Mazzanti M., Florio T. (2019). Emerging Role of Cellular Prion Protein in the Maintenance and Expansion of Glioma Stem Cells. Cells.

[25] Romano A., Palizzi S., Romano A., Moltoni G., Di Napoli A., Maccioni F., Bozzao A. (2023). Diffusion Weighted Imaging in Neuro-Oncology: Diagnosis, Post-Treatment Changes, and Advanced Sequences—An Updated Review. Cancers.

[26] Zhao Y., Xu J., Chen B., Cao L., Chen C. (2022). Efficient Prediction of Ki-67 Proliferation Index in Meningiomas on MRI: From Traditional Radiological Findings to a Machine Learning Approach. Cancers.

[27] Ravnik J., Rowbottom H. (2024). The Impact of Molecular and Genetic Analysis on the Treatment of Patients with Atypical Meningiomas. Diagnostics.

